# Arrivals: tell the migration with images

**DOI:** 10.3402/ejpt.v4i0.22986

**Published:** 2013-12-05

## XIII ESTSS Conference: “Trauma and its clinical pathways: PTSD and beyond”, Bologna, June 2013 ORAL, JUNE 8 HALL SYDNEY

## Open Papers: Contesti traumatici complessi

### “Approdi: narrare la migrazione per immagini” 16.00–16.15

#### Diego Manduri Istituto di Terapia Familiare di Bologna

Partendo dalla difficoltà di dare supporto e ascolto ai migranti, alle loro storie e al loro dolore, è stata creata e sperimentata una tecnica di narrazione che partisse dalle suggestioni dell'immagine. Integrando due aspetti esemplificativi della formazione e della pratica clinica dell'istituto di terapia familiare di Bologna: il pensiero sistemico-relazionale e l'uso delle immagini, è stato creato uno strumento narrativo ad-hoc usando la graphic-novel “l'approdo” di Shuan Tan. Le immagini altamente suggestive vengono viste, scelte e poi narrate all'interno di un gruppo di migranti che hanno condiviso un percorso di insegnamento della lingua. Abbiamo sperimentato “Approdi”, in 5 classi di un corso di italiano per stranieri, per un totale circa di 100 migranti. I risultati della sperimentazione confermano le facilitazioni che l'immagine da nel rievocare, narrare, accogliere, comprendere e contenere storie complesse, ricche e dolorose.

### Arrivals: tell the migration with images

Starting from the difficulty of giving support and consultation to migrants on their stories and pain, a technique of storytelling that departed from the suggestions of the image has been created and tested. By integrating two aspects of training, the systemic-relational thinking and the use of images, at the Institute of Family Therapy of Bologna, we created a narrative tool *ad hoc* using “The Arrival”, a graphic novel by Shuan Tan. The images are highly suggestive views, choices, and then narrated within a group of migrants who have shared a path of teaching. We tested “Arrivals” in five classes of an Italian course for a total of about 100 migrants. The experimental results confirm that the image facilitates recall, recount, accept, and understand complex, rich, and painful stories.

